# Foot care behaviours and associated factors among patients with type 2 diabetes: A cross-sectional study

**DOI:** 10.7189/jogh.14.04145

**Published:** 2024-08-23

**Authors:** Chin-Siang Ang, Kelley Fann Ing Goh, Nandika Lodh, Vicky Mengqi Qin, Huiling Liew, Harvinder Raj Singh Sidhu, Jun Jie Ng, Tavintharan Subramaniam, Elaine Tan, Gerald Choon Huat Koh, James Best, Julian Wong, Josip Car, Andy Hau Yan Ho, Kavita Venkataraman

**Affiliations:** 1 Centre for Population Health Sciences, Lee Kong Chian School of Medicine, Nanyang Technological University, Singapore, Singapore; 2 Department of Endocrinology, Tan Tock Seng Hospital, Singapore, Singapore; 3 Department of Surgery, Ng Teng Fong General Hospital, Singapore, Singapore; 4 Division of Vascular and Endovascular Surgery, Department of Cardiac, Thoracic and Vascular Surgery, National University Heart Centre, Singapore, Singapore; 5 Department of Surgery, Yong Loo Lin School of Medicine, National University of Singapore, Singapore, Singapore; 6 Diabetes Centre, Admiralty Medical Centre (AdMC), Singapore, Singapore; 7 Toa Payoh Polyclinic, National Healthcare Group Polyclinics, Singapore, Singapore; 8 Saw Swee Hock School of Public Health, National University of Singapore, Singapore, Singapore; 9 Lee Kong Chian School of Medicine, Nanyang Technological University, Singapore, Singapore; 10 Department of Cardiac, Thoracic and Vascular Surgery, National University Hospital, Singapore, Singapore; 11 School of Life Course and Population Sciences, King's College London, Strand, London, UK; 12 Psychology Programme, School of Social Sciences, Nanyang Technological University, Singapore, Singapore

## Abstract

**Background:**

As numerous studies highlighted the importance of maintaining proper foot care (FC) behaviours among individuals with diabetes to prevent complications, we sought to assess FC behaviours among patients with diabetes and to identify the factors associated with the practice of diabetic FC.

**Methods:**

We used a cross-sectional design and collected data through self-reported questionnaires administered to a sample of 586 patients from five medical centres. We conducted descriptive and inferential analyses to explore the relationships between potential risk and protective factors and FC behaviours.

**Results:**

Overall, 429 individuals (73.2%) had good FC behaviours, while 157 (26.8%) displayed poor FC behaviours. Furthermore, we identified eight influencing factors on FC behaviours, including smoking status, the availability of a caregiver, the presence of diabetic foot ulcers, amputation history, FC knowledge, subjective norms in diabetes self-care behaviour, diabetes-related stress, and quality of life index values. The logistic regression analysis showed that current smokers were 60% less likely to practice good FC compared to non-smokers (odds ratio (OR) = 0.40; 95%; confidence interval (CI) =  0.22–0.73). Having a caregiver decreased the likelihood of practicing good FC by 50% (OR = 0.52; 95% CI = 0.33–0.84), while having diabetic foot ulcers doubled it (OR = 2.65; 95% CI = 1.26–5.54). Additionally, more FC knowledge increased the likelihood by 20% (OR = 1.21; 95% CI = 1.10–1.33), and higher diabetes-related stress increased it by 1.03 times (OR = 1.03; 95% CI = 1.02–1.05).

**Conclusions:**

Our findings underscore the interplay of various factors influencing FC behaviours among individuals with diabetes and call for targeted interventions and tailored strategies to improve FC practices in this vulnerable population.

Type 2 diabetes mellitus (T2DM) is a highly prevalent chronic disease and the ninth leading cause of mortality worldwide [1]. An estimated 529 million people had diabetes in 2021, with T2DM accounting for approximately 96% of all diabetes cases globally [2]. The prevalence of T2DM is particularly pronounced in Asia, with 60% of people with diabetes (PWD) originating from this region [3]. In Singapore, projections indicate that T2DM prevalence will increase from 7.3% in 1990 to 15% by 2050 [4].

A consequence of T2DM is the increased vulnerability of individuals to developing various complications, with diabetic foot ulcers (DFUs) being particularly common within the population of diabetes [5]. DFUs are defined by the International Working Group on the Diabetic Foot as full-thickness wounds extending through the dermis, located below the ankle in PWD [6]. According to a systematic review, the global prevalence of DFUs stands at 6.3% [7], while up to 34% of PWD will likely experience DFUs over their lifetime [8]. In Singapore, the incidence of initial DFUs is 28.29 per 1000 person-years, with 5.8% of affected individuals subsequently requiring an amputation [9]. The country also had one of the highest rates of lower extremity amputations globally in 2016 [10], which have remained consistently high over time [11].

DFUs contribute significantly to increased risk of hospital admissions, amputations, and mortality in diabetes [12,13]. A 10-year retrospective study involving 2170 PWDs conducted by the National Health Group, Singapore, found that individuals who underwent lower extremity amputation had an average age of 64 years, with 22% of them passing away within one year following the amputation [14]. Moreover, recurrent foot ulcerations have negative effects on patients' physiological well-being, mental health, and social functioning [15], while also contributing significantly to their medical burden due to ongoing costs associated with wound management [16].

Preventing diabetic foot complications and amputations requires the adoption of regular foot care (FC) practices. Evidence-based diabetic FC behaviours have proven to be a cost-effective and essential primary preventive measure for DFUs and related complications [17,18]. They encompass a range of practices, including foot inspection, foot hygiene, toenail care, basic wound management, and proper footwear selection. Adherence to these practices enables early detection and intervention for DFUs.

FC adherence, in general, is a complex behaviour, with influencing factors varying based on individuals’ attitudes, knowledge, social and economic status, disease characteristics, and the type of treatment [19,20]. A recent review highlighted several determinants influencing PWDs' FC behaviours, such as demographic characteristics, FC education, knowledge about FC, and illness beliefs and perceptions [21]. For example, female patients, those with higher education levels, higher socioeconomic status, better FC knowledge, stronger illness beliefs and perceptions, and those who receive adequate support for education and information have been reported to engage more frequently in FC. Despite these factors, most studies included in the aforementioned review indicated that patients' FC behaviour remains suboptimal. Given the high rates of DFU and lower extremity amputation in Singapore, there is a need to recognise the barriers and facilitators that influence adequate FC in PWD.

We aimed to investigate the current status of FC behaviours among patients with T2DM and analyse the influencing factors. Our findings could provide valuable insights into the decision-making processes of patients regarding adherence to FC, ultimately helping prevent future complications.

## METHODS

### Study design and setting

This was a multicentre cross-sectional study carried out in various hospitals and polyclinics across Singapore, including the National University Hospital, the Ng Teng Fong General Hospital, the Tan Tock Seng Hospital, the Admiralty Medical Center, and the National Healthcare Group Polyclinic Toa Payoh, between February and June 2023. We obtained ethical approval for this study from the Research Ethics Committee of the National Healthcare Group (DSRB Ref: 2021/00618) and the Institutional Research Board of the Nanyang Technological University (IRB-2022-109). We followed the Declaration of Helsinki in conducting this study and the Strengthening the Reporting of Observational Studies in Epidemiology (STROBE) guidelines [22] in presenting our findings.

### Sample size

We determined the minimum sample size to be 551 patients, considering 10 predictive variables, an effect size (f2) of 0.03, a significance level of 0.05, a power of 0.80, and the use of regression as the analytical strategy to determine the factors associated with FC behaviour [23]. Given that most PWD are elderly, we estimated a 5% proportion of incomplete responses. Therefore, we set the final sample size at 580 patients.

### Inclusion and exclusion criteria

To be eligible, participants had to be Singaporean or Singapore permanent resident; diagnosed with T2DM; have T2DM for at least three months; be aged 21 years or older; speak and read either English, Mandarin, or Malay; be able to give informed consent; and be inpatients or outpatients. We excluded participants who had severe mental illnesses, such as psychosis and alcohol dependency; lacked the capacity for self-care; had cognitive impairments; were illiterate; experienced severe communication difficulties; refused to provide consent; had a history of bilateral below-knee/above-knee amputation; or were pregnant women.

### Procedure

Healthcare professionals at each study site were responsible for identifying eligible participants, approaching them, and inviting them to participate. Participation in the study was entirely voluntary, and those who agreed to take part completed an informed consent form. Subsequently, we provided participants either with an online questionnaire or a hard copy for those unwilling or unable to complete the survey online due to issues with internet connectivity or insufficient technical skills. Throughout the survey process, researchers were available to promptly address and clarify any confusion arising from the questions. Participants completing the survey received a SGD 25 National Trades Union Congress FairPrice voucher as a token of our appreciation for their time.

### Measures

We collected the data for this study using a structured, pre-tested questionnaire developed based on previously published measures [24–29]. The questionnaire was initially prepared in English and then translated into two commonly spoken local languages: Mandarin and Malay. To maintain linguistic accuracy and conceptual relevance in the translated versions, we enlisted professional translators for the translation and engaged experts in clinical practice in consensus meetings to review and verify its accuracy. We conducted a pilot study on 30 participants to assess the questionnaire's appropriateness and gather feedback.

We assessed the outcome of FC behaviour with the Nottingham Assessment of Functional Footcare Revised 2015 (NAFF-R) [24], a self-reporting-based questionnaire consisting of 26 items that assess the extent to which individuals follow recommended FC behaviours. Participants rated each item on a scale from 0 to 3 (e.g. ‘never’ to ‘often’ or ‘once a week or less’ to ‘more than once a day’) based on the frequency of the behaviour. We opted to use 23 relevant items following feedback and expert input that three items (‘Do you use a hot water bottle in bed’, ‘Do you put your feet near the fire’, and ‘Do you put your feet on a radiator’) were not applicable in the Singapore context. We summed the scores from these items to create a total score, which ranged from 0 to 69, with higher scores indicating better FC behaviour. We established a cutoff point of ≤50% [30] to identify individuals with poor FC behaviour, while scores above 50% indicated good FC behaviour.

The Questionnaire of Foot Care Knowledge (FCK) [25] comprises 15 items designed to assess individuals' understanding of FC. Each item had ‘true,’ ‘false,’ and ‘do not know’ response options; a correct response is scored as 1 point, while other responses receive 0 points. The total score ranged from 0 to 15, with higher scores indicating greater FC knowledge.

The Diabetes Intention, Attitude, and Behaviour Questionnaire (DIAB) [26] scale includes 17 items, covering six psychological constructs related to diabetes self-care: subjective norm, attitude, perceived behavioural control, intention, planning, and behaviour. Items are rated on a seven-point (e.g. ‘strongly disagree’ to ‘strongly agree’ or ‘not at all valuable’ to ‘extremely valuable’) or eight-point scale (‘0 days’ to ‘every day’). We summed the scale scores subscales to create separate subscores, with higher scores indicating higher levels of engagement in diabetes self-care behaviour.

The Problem Areas in Diabetes (PAID) [27] scale includes 20 items that measure emotional distress in people with diabetes. Responses are rated on a five-point Likert scale from 0 (not a problem) to 4 (serious problem), with scores ranging from 0 to 80, whereby higher scores indicate more severe emotional distress.

The Mutuality Scale (MS) [28] has 15 items which measure overall mutuality from the patient's perspective and its four domains (i.e. reciprocity, love and affection, shared pleasurable activity, and shared values). To reduce the survey burden on the participants, we took all items from the two domains within the scale (shared values (two items) and reciprocity (six items), for a total of eight items. The items themselves are rated on a five-point Likert scale from 0 (not at all) to 4 (a great deal), with the total score ranging from 0 to 32 and higher scores indicating greater mutuality.

The European Five-Dimensional Health Scale (EQ-5D-5L) [29] assesses health-related quality of life across five dimensions: mobility, self-care, usual activities, pain or discomfort, and anxiety or depression. Items are measured using a five-point Likert scale, with responses ranging from ‘no problems’ to ‘unable to/ extreme problems.’ We used the eq5d command to derive health utility index values from individual responses to the EQ-5D-5L. The EQ-5D-5L index has an upper bound equal to 1 which indicates full health (as evidenced by ‘no problem’ in all dimensions), while 0 represents death [31]. Additionally, the EQ-5D-5L includes a visual analogue scale for overall health status, ranging from 0 (worst) to 100 (best).

### Participants’ general characteristics

We collected participants self-reported information on various sociodemographic and medical history characteristics. The sociodemographic variables included age, sex, nationality, ethnicity, marital status, education status, occupational status, monthly household income in SGD, living arrangement, type of dwellings, smoking status, and availability of a carer. We categorised sex as male or female; nationality as Singaporean or permanent resident; and ethnicity as Chinese, Malay, Indian, and others. Marital status included non-married (including widowed, divorced, or never married) and married. We grouped education status into primary education and below, secondary education, and post-secondary education. We classified occupational status as working or not working. We divided monthly household income into two levels: less than SGD 2000 and  equal to or above SGD 2000, to align with previous local studies defining low-income households as those earning less than SGD 2000 per month [32,33]. We categorised living arrangement as living with others or living alone, and the type of dwellings as Housing & Development Board flat or private properties. We classified smoking status as never smoker, former smoker, or current smoker. We determined availability of a carer by the question ‘Do you receive care and support from family members/friends or a domestic home worker due to your health condition?’, with responses being ‘yes’ or ‘no’. For medical history, we collected information regarding the duration of diabetes, present diabetes treatment (which included oral medication or oral medication + insulin, in addition to diet control), history of DFU (never, had, or currently having DFU; considering the data distribution, we collapsed the ‘had/ have DFU’ categories for inferential statistics), and history of amputation (‘yes’ or ‘no’). We analysed these characteristics as exposure variables.

### Statistical analysis

Prior to the analysis, we conducted normality tests by assessing the skewness and kurtosis values, whereby values falling within two standard deviations (SDs) are considered normally distributed [34]. We presented continuous variables as means (x̄) and SDs (normal distribution) or medians (MDs) and interquartile ranges (IQRs) (skewed distribution) and categorical variables as numbers and proportions. To assess statistical differences between those with poor and good FC behaviours, we used independent sample *t* tests (normal distribution) or Mann-Whitney U tests (skewed distribution) for continuous variables, and χ^2^ tests for categorical variables. We then conducted a multivariate logistic regression analysis to identify potential protective and risk factors associated with FC behaviour among the participants. We considered variables with a *P*-value ≤0.05 in the bivariate analysis as candidates for inclusion in the multivariable analysis. We presented the results of this analysis as odds ratios (ORs) and 95% confidence intervals (CIs). We defined statistical significance as a *P*-value <0.05.

We used SPSS version 22 (IBM Corp., Armonk, NY, USA) for all statistical analyses.

## RESULTS

### Overall sample characteristics

Out of the 586 participants in the study, 55.5% were male and 44.5% were female (**Table 1**). Around 97% identified themselves as Singaporean nationals, while 45.2% identified as Chinese. In terms of marital status, 395 (67.4%) participants reported being married. Regarding educational backgrounds and occupation, 232 participants (39.6%) had 'secondary education', while 385 (65.7%) to as non-working. Similarly, 365 (62.4%) participants reported a monthly household income of less than SGD 2000. Concerning living arrangements and dwelling type, 530 (90.4%) participants reported 'living with others', while 546 (93.3%) indicated they resided in a Housing & Development Board flat. Further, 418 participants (71.3%) were never smokers, while 417 (71.2%) reported receiving care from their carers due to health reasons. The average reported duration of diabetes was 16.17 years (SD = 12.05), while 333 participants (56.8%) reported using oral medication for the treatment of diabetes. In terms of the presence of DFU and amputation history, 149 participants (25.4%) reported a history of DFU, while 119 (20.3%) had experienced amputation.

**Table 1:** Overall sample characteristics (n = 586)

Variables by category	n (%)*
Age in years (n = 585), x̄ (SD)†	63.82 (12.44)‡
Sex	
*Male*	325 (55.5)
*Female*	261 (44.5)
Nationality	
*Singaporean*	569 (97.1)
*Permanent resident*	17 (2.9)
Ethnicity	
*Chinese*	265 (45.2)
*Malay*	180 (30.7)
*Indian and others*	141 (24.1)
Marital status	
*Non-married*	191 (32.6)
*Married*	395 (67.4)
Education status	
*Primary education and below*	185 (31.6)
*Secondary education*	232 (39.6)
*Post-secondary education*	169 (28.8)
Occupational status	
*Working*	201 (34.3)
*Not working*	385 (65.7)
Household income (n = 585)†	
*<SGD 2000*	365 (62.4)
*≥SGD 2000*	220 (37.6)
Living arrangement	
*Living with others*	530 (90.4)
*Living alone*	56 (9.6)
Type of dwelling (n = 585)†	
Housing & Development Board flat	546 (93.3)
Private properties	39 (6.7)
Smoking status	
Never smoker	418 (71.3)
Former smoker	98 (16.7)
Current smoker	70 (12)
Availability of a carer	
*No*	169 (28.8)
*Yes*	417 (71.2)
Duration of diabetes, x̄ (SD)	16.17 (12.05)§
Present diabetes treatment	
*Oral medication*	333 (56.8)
*Oral medication and insulin*	253 (43.2)
Presence of diabetic foot ulcer	
*Never*	437 (74.6)
*Had*	22 (3.7)
*Have*	127 (21.7)
Amputation history	
*No*	467 (79.7)
*Yes*	119 (20.3)

SD – standard deviation, x̄ – mean

*Values presented as n (%) unless otherwise specified.

†No missing values of included variables unless otherwise noted.

‡Normality test for age was −0.48 (skewness) and 0.56 (kurtosis).

§Normality test for duration of diabetes was 0.92 (skewness) and 0.35 (kurtosis).

### FC behaviour

Applying a cutoff score of 50%, 429 (73.2%) participants had good FC practices, while 157 (26.8%) showed suboptimal practices. Examining specific aspects (**Table 2**), 348 (59.4%) participants reported inspecting their feet at least once daily. Furthermore, 114 (19.5%) participants did not check their shoes before putting them on, and 155 (26.5%) skipped checking their shoes when taking them off. Regarding foot hygiene, 546 (93.1%) participants washed their feet at least once daily. After washing, 193 (33.0%) of the participants rarely or never checked if their feet were dry, and 218 (37.2%) seldom or never dried their toes. For moisturizer use, 116 (19.8%) participants did not apply it to their feet, while 33.3% applied it between their toes multiple times a day.

**Table 2:** Distribution of questionnaire responses related to foot care behaviour, presented as n (%)

Item	Once a week or less	2–6 times a week	Once a day	More than once a day
Do you examine your feet?	120 (20.5)	70 (11.9)	234 (39.9)	162 (27.6)
Do you wash your feet?	7 (1.2)	33 (5.6)	179 (30.5)	367 (62.6)
**Item**	**Never**	**About once a month**	**Once a week**	**Daily**
Do you use moisturizing cream on your feet?	116 (19.8)	41 (7)	158 (27)	271 (46.2)
Do you put moisturizing cream between your toes?	218 (37.2)	49 (8.4)	124 (21.2)	195 (33.3)
**Item**	**Never**	**Less than once a month**	**About once a month**	**About once a week**
Are your toenails cut?	25 (4.3)	39 (6.7)	205 (35)	317 (54.1)
**Item**	**<4 times a week**	**4–6 times a week**	**Daily**	**More than once a day**
Do you change your socks/stockings/tights?	232 (39.6)	95 (16.2)	239 (40.8)	20 (3.4)
**Item**	**Rarely/never**	**Sometimes**	**Often**	**Always**
Do you dry between your toes?	218 (37.2)	115 (19.6)	77 (13.1)	176 (30)
Do you break into new shoes gradually?	155 (26.5)	44 (7.5)	21 (3.6)	366 (62.5)
**Item**	**Never**	**Rarely**	**Sometimes**	**Often**
Do you check your shoes before you put them on?	114 (19.5)	91 (15.5)	182 (31.1)	199 (34)
Do you check your shoes when you take them off?	155 (26.5)	164 (28)	164 (28)	103 (17.6)
Do you check your feet are dry after washing?	86 (14.7)	107 (18.3)	154 (26.3)	239 (40.8)
Do you wear shoes without socks/stockings/ tights?	146 (24.9)	58 (9.9)	143 (24.4)	239 (40.8)
Do you walk around the house in bare feet?	77 (13.1)	47 (8)	87 (14.8)	375 (64)
Do you walk outside in bare feet?	407 (69.5)	123 (21)	34 (5.8)	22 (3.8)
Do you use corn remedies/ corn plasters/ paints when you get corn?	256 (43.7)	99 (16.9)	154 (26.3)	77 (13.1)
Do you put a dry dressing on a blister when you get one?	186 (31.7)	106 (18.1)	178 (30.4)	116 (19.8)
Do you put a dry dressing on a graze, cut, or burn when you get one?	164 (28)	101 (17.2)	195 (33.3)	126 (21.5)
**Item**	**Never**	**Rarely**	**Sometimes**	**Most of the time**
Do you wear slippers with no fastening?	92 (15.7)	31 (5.3)	27 (4.6)	436 (74.4)
Do you wear trainers?	315 (53.8)	86 (14.7)	111 (18.9)	74 (12.6)
Do you wear shoes with lace-up, Velcro or strap fastening?	283 (48.3)	88 (15)	120 (20.5)	95 (16.2)
Do you wear pointed-toed shoes?	474 (80.9)	77 (13.1)	28 (4.8)	7 (1.2)
Do you wear flip-flops or mules?	383 (65.4)	80 (13.7)	84 (14.3)	39 (6.7)
Do you wear artificial fibre (e.g. nylon) socks?	252 (43)	135 (23)	119 (20.3)	80 (13.7)

*No missing values of included variables unless otherwise noted.

We also found that 269 (45.9%) participants trimmed their toenails once a month or less frequently. In terms of footwear, 436 (74.4%) participants preferred slip-on shoes. Over half of the participants reported never wearing athletic trainers (n = 315, 53.8%), pointed-toed shoes (n = 474, 80.9%), or flip-flops/mules (n = 383, 65.4%). Moreover, 239 (40.8%) participants often went sockless with their shoes, and 232 (39.6%) changed socks less than four times a week. Regarding barefoot walking, 375 (64%) participants did so indoors, while 22 (3.8%) did so outdoors. Finally, we identified that 256 (43.7%) participants did not use corn remedies, corn plasters, or paints, while 186 (31.7%) did not apply any dry dressing to a blister and 164 (28%) skipped dry dressing for grazes, cuts, or burns.

### The association between study variables and FC behaviour

Participants with poor FC behaviour were more likely to be current smokers (17.8% vs 9.7%; *P* < 0.05) and gave carers present (78.3% vs 68.5%; *P* < 0.05). Conversely, those reporting good FC behaviours were more likely to have DFU (31% vs 10.2%; *P* < 0.001) and a history of amptuations (25.2% vs 7%; *P* < 0.001). A post-hoc χ^2^ analysis showed a significant association between a history of DFU and amputations, with amputations largely occurring within the subset of individuals with a DFU history (n/N = 100/149, 67%; χ^2^ = 270.5; *P* < 0.001). Moreover, participants with higher FC knowledge, lower levels of subjective norms, higher diabetes-related stress, and a lower quality of life index value, generally exhibited good FC behaviour (**Table 3**).

**Table 3:** The results of the association between study variables and foot care behaviour*

	Foot care behaviour†			
**Category by variable**	**Poor (n = 157)**	**Good (n = 429)**	**Z/χ^2^**	***P*-value**
Age in years, x̄ (SD)	65 (56–72)‡	65 (56–72)	–0.23§	0.820
Sex				
*Male*	78 (49.7)	247 (57.6)	2.90¶	0.089
*Female*	79 (50.3)	182 (42.4)		
Nationality				
*Singaporean*	152 (96.8)	417 (97.2)	0.06¶	0.804
*Permanent resident*	5 (3.2)	12 (2.8)		
Ethnicity				
*Chinese*	73 (46.5)	192 (44.8)	2.38¶	0.304
*Malay*	53 (33.8)	127 (29.6)		
*Indian and others*	31 (19.7)	110 (25.6)		
Marital status				
*Non-married*	57 (36.3)	134 (31.2)	1.35¶	0.145
*Married*	100 (63.7)	295 (68.8)		
Education status				
*Primary education and below*	58 (36.9)	127 (29.6)	4.57¶	0.102
*Secondary education*	63 (40.1)	169 (39.4)		
*Post-secondary education*	36 (23)	133 (31)		
Occupational status				
*Working*	50 (31.8)	151 (35.2)	0.57¶	0.449
*Not working*	107 (68.2)	278 (64.8)		
Household income				
*<SGD 2000*	96 (61.5)‡	269 (62.7)	0.07¶	0.797
*≥SGD 2000*	60 (38.5)‡	160 (37.3)		
Living arrangement				
*Living with others*	140 (89.2)	390 (90.9)	0.40¶	0.526
*Living alone*	17 (10.8)	39 (9.1)		
Type of dwellings				
*Housing & Development Board flat*	150 (96.2)‡	396 (92.3)	2.72¶	0.099
*Private properties*	6 (3.8)‡	33 (7.7)		
Smoking status				
*Never smoker*	108 (68.8)	310 (72.3)	7.86¶	0.020
*Former smoker*	21 (13.4)	77 (17.9)		
*Current smoker*	28 (17.8)	42 (9.8)		
Availability of a carer				
*No*	34 (21.7)	135 (31.5)	5.39¶	0.020
*Yes*	123(78.3)	294 (68.5)		
Duration of diabetes, x̄ (SD)	13 (6–23)	13 (6–22)	−0.17§	0.863
Present diabetes treatment				
*Oral medication*	93 (59.2)	240 (55.9)	0.51¶	0.476
*Oral medication and insulin*	64 (40.8)	189 (44.1)		
Presence of diabetic foot ulcer				
*Never*	141 (89.8)	296 (69)	26.25¶	0.000
*Had/have diabetic foot ulcer*	16 (10.2)	133 (31)		
Amputation history				
*No*	146 (93)	321 (74.8)	23.44¶	0.000
*Yes*	11 (7)	108 (25.2)		
Foot care knowledge	10 (8–10.5)	10 (9–11)	−4.28§	0.000
Subjective norm	18 (16–19)	17 (14–19)	−2.15§	0.031
Attitude	18 (15.5–19)	18 (15–20)	−0.30§	0.768
Perceived behavioural control	18 (16–19)	18 (15–19)	−1.70§	0.089
Intention	17 (14–20)	17 (14–19)	−1.10§	0.277
Planning	17 (14.50–19)	17 (14–19)	−0.94§	0.347
Behaviour	11 (7.5–13.5)	11 (7–13)	−1.53§	0.127
Diabetes-related stress	7 (3–16)	19 (6–36)	−6.42§	0.000
Mutuality	29 (2.5–32)	26 (0–32)	−1.46§	0.145
Quality of life index value	1 (0.77–1)	0.81 (0.71–1)	−3.59§	0.000
Self-rated health	70 (55–80)	70 (51–80)	−0.54§	0.587

SD – standard deviation, x̄ – mean

*Values presented as n (%) unless otherwise specified. No missing values of included variables unless otherwise noted.

†Normality test for foot care practice was -0.01 (skewness) and 2.79 (kurtosis).

‡n = 156.

§Mann-Whitney U-test.

¶Pearson chi-Square.

### Multivariate logistic regression analysis

Building on the preceding analysis, we conducted a logistic regression (**Table 4**) to explore the influence of several factors – smoking status, availability of a caregiver, presence of DFU, history of amputation, FC knowledge, subjective norm, diabetes-related stress, and quality of life index value – on the likelihood of participants engaging in FC behaviour (categorised as either poor or good). The logistic regression model accounted for 23.8% (Nagelkerke R^2^) of the variation in FC behaviour and accurately classified 74.7% of the cases.

**Table 4:** The results of multivariate logistic regression analysis

Category by variable	β	SE	OR (95% CI)	*P*-value
Smoking status				
*Never smoker*	ref	ref	ref	ref
*Former smoker*	0.16	0.29	1.12 (0.66–2.09)	0.576
*Current smoker*	−0.92	0.31	0.40 (0.22–0.73)	0.003
Availability of a carer				
*No*	ref	ref	ref	ref
*Yes*	−0.65	0.24	0.52 (0.33–0.84)	0.007
Presence of diabetic foot ulcer				
*No*	ref	ref	ref	ref
*Yes*	0.97	0.38	2.65 (1.26–5.54)	0.010
Amputation history				
*No*	ref	ref	ref	ref
*Yes*	0.65	0.43	1.92 (0.82–4.49)	0.134
Knowledge	0.19	0.05	1.21 (1.10–1.33)	0.000
Subjective norm	−0.03	0.03	0.97 (0.91–1.03)	0.317
Diabetes-related stress	0.03	0.01	1.03 (1.02–1.05)	0.000
Quality of life index value	−0.30	0.62	0.74 (0.22–2.48)	0.623
Constant	−0.27	0.95	0.77	0.779

CI – confidence interval, OR – odds ratio, SE – standard error, β – logistic regression coefficient

The odds of good foot care practice decreased by 60% for current smokers compared to never smokers (OR = 0.40; 95% CI = 0.22–0.73). Those participants who had a caregiver were half as likely to engage in good FC behaviours compared to those without a caregiver (OR = 0.52; 95% CI = 0.33–0.84). We found the largest association between the presence of DFU (participants with either a current active ulcer or a history of ulcer) and FC behaviours, where the presence of DFU was associated with a 2-fold increase in the likelihood of good FC behaviour (OR = 2.65; 95% CI = 1.26–5.54).

Furthermore, participants with good FC knowledge were about 20% more likely to practice good FC (OR = 1.21; 95% CI = 1.10–1.33). We also observed a link between diabetes-related stress and FC behaviours. with the odds of exhibiting good FC practices increasing 1.03 times with each unit increase in the diabetes-related stress score (OR = 1.03; 95% CI = 1.02–1.05). However, the remaining predictors – amputation history, subjective norm, and quality of life index value – were not statistically significant.

## DISCUSSION

We investigated FC behaviours in patients with T2DM and explored factors influencing these practices. Our findings showed that a substantial proportion of participants adhered to recommended FC practices like daily examinations, frequent washing, and regular toenail trimming. However, we also saw a need for improvement in certain areas. A significant proportion of participants reported suboptimal behaviour, such as walking barefoot indoors, neglecting to dry between toes, and frequently choosing slippers. These practices can increase susceptibility to foot injuries and infections [35–37]

Suboptimal FC practices in these areas may be attributed to a lack of education about associated risks and the importance of these practices in preventing foot complications [38]. Additionally, the preference for walking barefoot at home and choosing slippers as footwear may be influenced by cultural and habitual practices. For example, many Asian households traditionally prefer not to wear footwear indoors to maintain cleanliness. Furthermore, the warm and humid climate in places like Singapore may inadvertently encourage the choice of less protective footwear options. This notion is supported by previous studies where patients have explained that the comfort, breathability, and sense of freedom offered by wearing slippers outdoors or going barefoot indoors are often favoured over more protective alternatives [39].

In the analysis, we found none of the sociodemographic factors to be significantly associated with FC behaviour. However, we identified eight predictors, five of which showed significant associations with FC behaviour in the multivariate model: smoking status, the presence of a caregiver, the presence of DFU, diabetes-related stress, and FC knowledge. Specifically, smoking emerged as a potential deterrent to good FC practices, whereby we found that current smokers were significantly less likely to engage in recommended FC routines than non-smokers. This trend may be rooted in the broader lifestyle choices and habits commonly associated with regular smokers. Existing studies have suggested that individuals who demonstrate non-adherence to one aspect of self-care, such as quitting smoking, may have a general inclination toward engaging in behaviours that are suboptimal for their health [40]. Smoking, a known risk factor for various health complications, may serve as an indicator of a lack of adherence to other health-promoting behaviours, including regular FC.

Contrary to previous research [41,42], the presence of caregivers was associated with lower FC behaviour scores in our study, whereby having a carer reduced the likelihood of good FC. This finding could be attributed to a reliance on caregivers, who may possess well-informed knowledge about proper FC practices. Patients might thus tend to delegate their responsibility of foot (self-) care entirely to their caregivers, rather than actively participating in their own FC regimen. This reliance on caregivers could result in a gap in personal accountability, potentially hindering the development of regular and informed FC habits among the patients themselves. Another plausible explanation could be that older patients face numerous self-care challenges due to age-related changes in health, social support, the presence of comorbidities, and physical and mental capabilities. Consequently, these older and sicker patients often become more dependent on caregivers, which may further hinder their ability to practice proper FC independently [43].

We found that the presence of DFUs increased the likelihood of good FC practices in participants. This might be attributed to a ‘wake-up call’ effect, where the severity of their conditions prompts individuals to become more attentive and cautious about their health. The finding is also consistent with prior research, which noted that patients experiencing symptoms of chronic heart failure tended to engage more in self-care behaviours [44]. This suggests a broader tendency among individuals dealing with serious health conditions to adopt more vigilant self-care practices as a reactive measure. The shift in behaviour can also be analysed using the Health Belief Model, a theoretical model explaining changes in health-related actions based on individuals’ health beliefs. Within our study, experiencing a DFU may have served as a trigger, prompting patients to reassess their perceived susceptibility and the severity of diabetic foot complications.

Moreover, we found that higher levels of diabetes-related stress marginally increased the likelihood of adhering to good FC practices. One possible explanation for this finding is that increased stress levels act as a constant reminder of the potential complications linked to diabetes. This heightened awareness may compel patients to be more self-conscious about their health, thereby fostering a greater commitment to adhering to proper FC practices [45].

Lastly, knowledge of proper FC emerged as a significant factor influencing FC behaviour. Participants with a stronger understanding of FC practices showed a significantly higher likelihood of adhering to recommended routines. This may be attributed to their enhanced awareness and knowledge regarding the crucial role of FC in managing diabetes. A body of prior research has consistently supported the correlation between knowledge gained and the improvement in FC behaviour [46,47]. Essentially, this knowledge serves as the cornerstone for FC behaviour because, without a well-rounded understanding, individuals are unlikely to engage in appropriate FC [48]. Unsurprisingly, studies have consistently underscored the significance of education as a potent preventive tool, where acquired knowledge directly translates into improved FC behaviour [47,49,50].

Meanwhile, we identified factors such as amputation history, subjective norms, and quality of life as significant predictors of FC behaviour in the bivariate analysis; however, they lost their predictive power when compared to others in the multivariate analysis. Several plausible explanations can account for this observation. First, amputation history may be closely related to the presence of DFU, as a post-hoc χ^2^ analysis found a significant association between a history of DFU and amputations. However, it seems that those with amputations were a subset of those with a DFU history, thereby overshadowing the unique contribution of amputation history when considered independently. Second, subjective norms, which encompass the social and cultural influences on an individual's behaviour, may be confounded by the presence of a caregiver, as they often play a significant role in a patient's social circle and are likely to influence their health behaviours. As such, when we include the variable of having a caregiver in our model, the predictive power of subjective norms may have diminished. Lastly, the quality of life may have been influenced by multiple factors within the model, such as the presence of DFU and diabetes-related stress. Both of these conditions can substantially impact a patient's quality of life, thereby rendering it a less robust independent predictor when considered alongside these other variables. Therefore, the reduced predictive power of these variables suggests that they may not independently influence FC behaviour; instead, they likely interact with other factors, diminishing their individual impact.

This study has several limitations. First, its cross-sectional design prevents us from establishing causality between the identified factors and FC behaviours. Longitudinal studies may offer deeper insights into causal relationships. Second, it was geographically confined to Singapore, which may limit the generalisability of the findings to populations with different sociocultural backgrounds. Furthermore, the reliance on self-reported data through questionnaires may have potentially introduced bias. Additionally, we used questionnaires adapted from overseas research that may not fully capture the unique aspects of FC behaviours within the specific context of Singapore. Future studies should address these limitations by adopting a more diverse methodological approach and broadening the geographical scope to increase the generalisability of their findings. Notably, we employed a modified version of the Mutuality Scale, utilising only two subscales and excluding aspects of love (e.g. ‘how much love do you feel for him or her?’) and shared pleasurable activities (e.g. ‘to what extent do you enjoy the time the two of you spend together?’). These elements might potentially be inapplicable to patient-carer mutuality, particularly when carers are hired helpers. While this tailoring enhanced contextual relevance, the decision to not use the full scale or all subscales might have impacted our results and interpretation of the questionnaire’s findings, or our understanding of the results in the broader context of mutuality as measured by the original instrument. Lastly, we used a financial incentive to enhance participation rates. The implemented approach, while effective, may have introduced an ‘incentive bias’, potentially affecting the data's integrity. However, the incentive amount was minimal and approved by an ethics board to mitigate this concern. Conversely, a lack of financial incentive might have introduced sampling bias, where participation would be limited to those who have the time or resources to contribute without compensation, potentially skewing the data towards a specific demographic. Future research should consider alternative recruitment methods and validation measures, like clinical examinations, to ensure a more accurate assessment of FC adherence.

### Implications for FC practice and research

Our insights into the various factors associated with FC behaviour have several implications for diabetic FC management and research. For example, we found that none of the sociodemographic factors were significantly associated with patient FC behaviour, suggesting limited disparities in non-modifiable factors among individuals in Singapore. In contrast, the determinants of managing diabetic foot issues, whether protective or risk factors, stem from factors that may be prevented or modifiable, such as physical and mental health problems related to diabetes, one’s level of FC knowledge, lifestyle choices, and the availability of caregivers. These factors present both areas of concern and opportunities for improving preventive FC practices.

Despite most participants reporting overall good FC practices, inequality in proper care of foot disease remains, and the practices related to different aspects of care vary among individuals. This variation highlights the need for a more comprehensive diabetes FC education program that addresses various aspects of FC and promotes uniform practices. Also, our finding that patients with the presence of DFU were more likely to have better FC behaviour raises concerns, as it suggests that experiencing symptoms might motivate patients to engage in more FC behaviour. However, patients with diabetic neuropathy may lose sensation associated with their ulcers, making it crucial to enhance patient education on diabetic foot disease symptoms beyond typical cues such as pain and discomfort. Future research should explore how FC behaviour changes over time following the implementation of a comprehensive educational intervention.

Related to the second implication, while our study focussed on the direct association between sociodemographic, physical, and psychological determinants of FC behaviour, it would be valuable to explore patients’ perceptions and beliefs regarding the cause of DFU. Qualitative research could improve our understanding of whether these perceptions play a role in the relationship between the severity of foot disease and FC practice. Such an approach would provide a nuanced understanding of the underlying drivers for their FC behaviour.

Another noteworthy observation is that the availability of caregivers was associated with poorer FC behaviour. This counterintuitive finding may be attributed to the fact that individuals who have caregivers may be in poorer health and less capable of taking responsibility for self-care. In many Asian societies, including Singapore, there is a strong tradition of caregiver support and caregivers often make significant sacrifices, such as reducing their other obligations and social activities to provide care [51,52]. This paradoxical situation highlights the complex interplay between the need for proper FC among diabetic patients and the potential impact on patient autonomy and caregiver burden. It remains essential to explore patients' expectations regarding the involvement of caregivers in the FC process and their perceptions of the responsibilities associated with self-care.

## CONCLUSIONS

Our findings highlight the continued need for improvement in FC practices among patients with T2DM. We recommend implementing regular screenings at specified intervals and targeted interventions for patients, and stress a need for designing carefully tailored support systems that do not over-support patients, but rather focus on equipping them with the necessary skills and knowledge to enhance their foot self-care management effectively.
